# Patient and provider attitudes toward genomic testing for prostate cancer susceptibility: a mixed method study

**DOI:** 10.1186/1472-6963-13-279

**Published:** 2013-07-20

**Authors:** Wendy C Birmingham, Neeraj Agarwal, Wendy Kohlmann, Lisa G Aspinwall, Mary Wang, Jay Bishoff, Christopher Dechet, Anita Y Kinney

**Affiliations:** 1 Department of Psychology, Brigham Young University, 1054 SWKT, Provo, UT 84602, USA; 2 Huntsman Cancer Institute, University of Utah, 2000 Circle of Hope, Salt Lake City, UT 84112, USA; 3 Department of Internal Medicine, University of Utah, 30 North 1900 East, Room 4C104, Salt Lake City, UT 84132, USA; 4 Department of Psychology, University of Utah, 380 South 1530 East, BEHS 502, Salt Lake City, UT 84112, USA; 5 Intermountain Health Care, 5169 Cottonwood St Ste 420, Murray, UT 84107, USA; 6 Department of Urology, University of Utah, 30 North 1900 East, Salt Lake City, 84132 UT, USA

**Keywords:** Genomics, Prostate cancer, Cancer screening, Attitudes, Providers

## Abstract

**Background:**

The strong association between family history and prostate cancer (PCa) suggests a significant genetic contribution, yet specific highly penetrant PCa susceptibility genes have not been identified. Certain single-nucleotide-polymorphisms have been found to correlate with PCa risk; however uncertainty remains regarding their clinical utility and how to best incorporate this information into clinical decision-making. Genetic testing is available directly to consumers and both patients and healthcare providers are becoming more aware of this technology. Purchasing online allows patients to bypass their healthcare provider yet patients may have difficulty interpreting test results and providers may be called upon to interpret results. Determining optimal ways to educate both patients and providers, and strategies for appropriately incorporating this information into clinical decision-making are needed.

**Methods:**

A mixed-method study was conducted in Utah between October 2011 and December 2011. Eleven focus group discussions were held and surveys were administered to 23 first-degree relatives of PCa patients living in Utah and 24 primary-care physicians and urologists practicing in Utah to present specific information about these assessments and determine knowledge and attitudes regarding health implications of using these assessments.

**Results:**

Data was independently coded by two researchers (relative Kappa = .88; provider Kappa = .77) and analyzed using a grounded theory approach. Results indicated differences in attitudes and behavioral intentions between patient and provider. Despite the test’s limitations relatives indicated interest in genetic testing (52%) while most providers indicated they would not recommend the test for their patients (79%). Relatives expected providers to interpret genetic test results and use results to provide personalized healthcare recommendations while the majority of providers did not think the information would be useful in patient care (92%) and indicated low-levels of genetic self-efficacy.

**Conclusions:**

Although similarities exist, discordance between provider and patient attitudes may influence the effective translation of novel genomic tests into clinical practice suggesting both patient and provider perceptions and expectations be considered in development of clinical decision-support tools.

## Background

Prostate cancer (PCa) is the most commonly occurring non-cutaneous cancer and the second leading cause of cancer deaths in American men [1]. In the U.S. in 2012, approximately 241,000 new cases of PCa were diagnosed and about 28,170 individuals died of the disease [2]. Having one first-degree relative with PCa at any age is associated with a twofold to threefold increased risk and risk increases with the number of first-degree relatives [3,4]. PCa risk is higher for men who have a brother with the disease than for those with an affected father [5]. The American Cancer Society recommends men discuss screening benefits and risks with their provider beginning at age 45 for men with a first-degree relative diagnosed with PCa < 65 years and at age 40 for men with two or more first-degree relative diagnosed with PCa < age 65 [5]. However, the U.S. Preventive Services Task Force recently released their recommendation against prostate-specific-antigen (PSA)-based screening for PCa [6] claiming that additional research is needed to determine the benefits and harms of PSA in men with a family history of the disease. These conflicting recommendations may leave men with a family history of PCa uncertain whether screening would be beneficial or harmful.

The strong association between family history and PCa risk suggests a significant genetic contribution, yet unlike breast and colorectal cancer in which highly penetrant predisposition genes have been found, specific PCa susceptibility genes have yet to be identified. Certain single nucleotide polymorphisms (SNPs) are correlated with PCa risk [7-9]. Risk estimates for individual SNPs have been associated with modest risk (OR ~ 1.1-1.8), but when inherited in combination the risk may be more pronounced [10]. Identification of these SNPs has led to the development of genomic PCa risk assessments. However, this testing is controversial as the functional significance of these individual genetic variants is still unknown and the clinical validity and utility of genomic testing for PCa risk assessment is yet undetermined. Such personalized approaches to PCa risk stratification are largely unexplored and formative research on attitudes, knowledge and interest in testing can help inform future public health and clinical communications as well as educational tools targeting both patients and providers.

Genomic testing for PCa susceptibility is available direct-to-consumers (DTC) through internet web sites (i.e., 23 and Me) and provider-ordered through a CLIA laboratory (ARUP). While this type of genomic testing is still controversial [11,12], both the public and healthcare providers are becoming more aware of its availability. Purchasing genomic risk assessments online allows patients to bypass their healthcare provider yet patients may have difficulty interpreting test results and may request providers help interpret results [13]. With increasing consumer interest in genomic testing [14,15] and evidence that providers are often not sufficiently educated in genetics to deliver clinical genetic services [16-18] there is a need to develop optimal strategies for appropriately educating patients and providers and to incorporate this information to inform health care decision-making. Understanding both patients’ and providers’ perspectives is crucial for effectively translating genomic discoveries into practice and developing effective decision support tools. Prior research has examined public awareness and perceptions of general DTC testing as well as provider knowledge and experience with general DTC testing [16,17,19]. To our knowledge, there are no available studies examining knowledge, attitudes, and interest in PCa genetic DTC and clinical genetic testing in men at increased familial risk for PCa and key providers who may order or be asked to interpret results from such tests.

The purpose of our study was to examine attitudes, knowledge, and behavioral intentions regarding genomic testing in relatives of men with PCa, and in providers. Urologists and primary care physicians are the most likely provider type to order genetic testing for PCa susceptibility or have patients ask them to interpret their DTC test results [20-22]. Thus, we specifically targeted these two subgroups. Our mixed-method approach included focus group discussions with relatives and providers to gain an understanding of decision support needs and inform the development of decision support tools^a^. We also administered quantitative surveys assessing cancer risk perceptions and genetic knowledge along with questions specifically developed to assess knowledge, attitudes about and interest in genomic testing.

## Methods

### Study design

Prior research indicates knowledge about and awareness of SNP testing is still fairly low in the general population. Therefore, to have a more informative discussion we provided a minimal level of background information through a brief education session that was conducted at the beginning of each focus group session [23]. The presentation for relatives included a description of well-established risk factors including age, race and family history, PCa screening guidelines, basic genetics (structure of DNA, explanation of SNPs), availability of SNP-based testing for PCa, possible test outcomes (including reviewing a sample report) and discussion of the benefits and limitations of SNP testing. The presentation for providers included information on SNP testing and validation of studies. While the study focuses on interest in a genomic test, the testing was described as genetic rather than genomic during the presentation as participants were more likely familiar with that terminology.

Additionally, SNPs occur at different frequencies in different racial populations. Therefore studies evaluating the role of SNPs in one population cannot necessarily be extrapolated to other racial groups. At the time of this study certain PCa tests were based on European populations. Because availability and accuracy of testing varies between racial and ethnic groups, it is important to seek out representation from diverse populations. Thus we stratified focus groups by race/ethnicity.

The use of focus groups is a well-validated methodology [24,25] and is particularly appropriate for examining translational issues regarding emergent technologies that are not yet routinely used in clinical practice [25]. Pre-focus group surveys for relatives assessed knowledge about and attitudes toward genetic testing, current PCa screening practices, knowledge of general genetics and genetic testing and standard demographic information. Pre-focus group surveys for providers assessed current patterns regarding referring, ordering, or explaining results of genetic testing, genetic self-efficacy and demographic information. Post-focus group surveys assessed attitudes toward the information presented during the focus group as well as providers’ likelihood of using genomic testing in clinical decision-making. The research protocol was reviewed and approved by the Internal Review Board of the University of Utah.

### Participants and setting

The relative subgroup consisted of 23 men aged 45–70 with at least one first-degree relative with PCa. Seventeen men had an affected father and 6 had an affected brother. Participants were recruited from the community through newspaper advertisements (*n*=7), local organizations (*n*=3), clinics at Huntsman Cancer Hospital (HCH) (*n*=8) and through the Utah site of the National Cancer Institute Cancer Genetics Network (CGN) (*n*=5) -a US national registry of individuals with a personal or family history of cancer [26]. Physicians from HCH clinics identified and gave permission to approach PCa patients. Study staff provided information about the study and requested permission to contact patients’ male unaffected first-degree relatives. Relatives were then sent a letter explaining the study, a consent letter and asked for permission to contact. CGN staff contacted unaffected relative enrollees to request permission for study staff to contact them about the study. Once permission to contact was obtained, staff screened the potential participant for eligibility over the phone, obtained consent participation and assigned participants to a focus group based on race/ethnicity. Relatives recruited through advertisements placed in a local newspaper and through community organizations were also screened for eligibility, consented and assigned to a focus group by phone.

Twenty-four providers were recruited through Huntsman Cancer Institute (*n*=6), community urology practices (*n*=8), and community practice clinics (*n*=10). Of these, 10 were primary care physicians and 14 were urologists or urology residents. Inclusion criteria for relative and provider groups included no personal history of cancer except non-melanoma skin cancers, no prior cancer risk counseling or evaluation and English fluency. All relative and provider participants who were screened eligible and assigned to a focus group completed the baseline survey, attended their assigned focus group and completed the post survey. Information on the composition of the focus groups is provided in Table 1.

**Table 1:** Relative and health care provider focus group composition

**Participants**	**Number of focus groups**	**Participants per focus group**	**Total N**
Relatives			
Non-Latino White	4	3-5	17
African American	1	3	3
Latino	1	3	3
Providers			
Urologists	1	9	9
Urology residents	1	5	5
Primary care physicians	3	2-5	10
Total	11		47

#### ***Measures***

Baseline and post focus group measures are presented in Additional file 1.

### Procedures

Relative focus groups were stratified by race/ethnicity. We held 4, 1 and 1 focus groups with non-Latino Whites, African-Americans, and Latinos, respectively. Provider focus groups were stratified by practice specialty with two urology focus groups and three primary-care focus groups. All focus groups were facilitated by the same moderator (WCB) and the educational presentation was given by a licensed genetic counselor (WK). Relative group discussions lasted approximately 120 minutes and provider group discussions lasted 90–120 minutes. All participants received compensation ($50 for relatives) or an honorarium ($150 or an Amazon Kindle e-reader for providers).

Pre-focus group surveys were mailed to participants and they returned the completed surveys at their assigned focus group meeting. Each focus group session began with the educational presentation. Information was presented on genetic testing and participants were shown a sample report from deCODE genetics, an online company that offered DTC testing at the time of this study^b^. The provider educational presentation included information on statistical calculations used to obtain risk estimates by online companies offering testing, and potential benefits and limitations of testing. Educational information presented to focus group participants is outlined in Additional file 2. Participants were then led in discussion by the moderator using a semi-structured moderator guide consisting of a series of open-ended questions. Immediately following the discussion participants completed post-focus group surveys. Focus groups were audio-recorded and transcribed verbatim by a professional transcription service and verified for accuracy by a research team member (MW).

### Data analysis

Descriptive analyses of questionnaire items were conducted using SAS 9.2 (SAS Institute Inc., Carry, NC, 2002–2010). Frequencies, percentages and measures of central tendency were calculated. NVivo9 software (QSR International Pty Ltd. Version 9, 2010) was used to code of focus group transcripts. All focus group transcripts were reviewed by research team members and a grounded theory approach [16,27,28] was used allowing codes, concepts and categories to emerge from the data. Our approach consisted of open coding of the data, organizing the data into segments based on key words and concepts to form categories and identify patterns and major themes in the focus group narratives. Interpretation of the data was discussed among the research team and all transcripts were then independently coded by MW and WCB line by line. Inter-rater reliability was determined by calculating Kappa statistics (relative transcripts = .88; provider transcripts = .77) and with overall percent agreement (98%).

## Results

Overall, no differences were found between the racial groups in their interest in and motivations for testing, thus groups were collapsed for analysis. Demographic information of relative and providers is presented in Table 2.

**Table 2:** Characteristics of study participants

		
**Relatives**		
Age, mean (standard deviation)	57 years (6.3)	
	n	%
Ethnicity/Race		
Non-Latino White	17	74%
African American	3	13%
Latino	3	13%
Employment		
For wages	18	78%
Self-employed	1	4%
Retired	4	18%
Have health insurance	22	96%
Income		
$30-49,999	1	4%
$50-69,999	6	26%
$70,000 or more	13	56%
Education level		
High school or GED	2	9%
Some college/AA, AS	9	39%
College graduate	7	30%
Postgraduate degree	5	22%
**Healthcare providers**		
Age, mean (standard deviation)	46 years (12.9)	
	n	%
Specialty		
Primary care	8	34%
Internal Medicine	2	8%
Urology	14	58%
Male	19	79%
Setting		
Primary practice	10	42%
Community urology	8	33%
Academic urology	6	25%
Years in practice		
≤10	11	46%
>10	13	54%

### Survey results

#### ***Relatives***

Baseline survey data indicated most relatives had not heard about DTC genetic testing (61%). Most relatives (67%) believed their own risk of PCa was great or very great and approximately half (56%) were somewhat or moderately worried about getting PCa. Fifty percent of relatives believed that genes determine how a person’s health behavior impacts his or her health. Relatives were divided with 35% agreeing or strongly agreeing, 43% disagreeing and 22% neither agreed nor disagreed that genes were more important than one’s own behavior in determining health. However, most (92%) believed that healthy behaviors could reduce the risk of disease for people who have a gene for a particular disease. Regarding PCa screening behavior, most relatives in our study had previously had a digital rectal exam (78%), 53% within the prior year. In addition, most had previously had their PSA levels tested (74%) with 63% reporting a PSA test within the prior year. Furthermore, nearly one-third of relatives had initiated conversations with their provider regarding cancer screenings (29%) because their provider had not brought it up, and more than half (55%) of relatives indicated their providers had not talked with them regarding their familial PCa risk.

Post-focus group survey data indicated most relatives (52%) were interested in obtaining the PCa SNP test and most (56%) would prefer to order it through their provider rather than on their own. Most relatives (96%) found the focus group presentation was helpful and free text comments indicated it was easy to understand and informational.

“…all of it was informational and easy to understand.”

“All was straight forward.”

Three relatives felt the lifetime risk estimates were difficult to understand and two relatives felt the presentation was biased toward getting the genomic testing; all other relatives indicated they liked the presentation and did not find the information too difficult to understand.

#### ***Providers***

Baseline survey data indicated that half of the providers had not heard or read about PCa DTC genetic testing. Most providers (92%) had not had a patient ask questions about DTC testing nor present results for interpretation. Although 42% of providers said they felt confident in their ability to assess risk of hereditary disorders, 92% did not feel confident discussing the benefits, risks and limitation of genetic testing with patients indicating low levels of provider genetic efficacy. Additionally, providers did not feel confident answering patients’ questions about DTC genomic tests (96%), did not feel prepared to answer patients’ questions about DTC genomic testing (79%) and did not feel confident in their knowledge of cancer genetics (74%). Providers were also concerned that increased-risk results could unnecessarily increase patient anxiety (74%). However, most agreed test results could help inform the age at which to start (86%) and frequency of recommended screenings (76%).

Post-focus groups surveys indicated most providers (92%) did not think SNP PCa test results would be useful in the management of patients, and most (79%) indicated they would not recommend SNP testing for PCa for their patients. Free text comments indicated providers believed SNP testing had too many limitations and was not practical or clinically useful.

“…Not sure if current tests are ready for prime time.” “…not validated by prospective studies.” “…not sensitive enough.” “…the cost-benefit value is suspect.”

“data is not compelling enough and seems more likely to lead to dilemma in interpretation and further recommendations.”

“It does not add any new information that PSA, history and digital rectal exam offers”

Free text comments also indicated that while overall the providers found the presentation clear and informative they wanted more detailed information on SNP testing validity.

### Focus group findings

Several themes emerged from the focus group discussions. The main themes are outlined in Table 3.

**Table 3:** Themes identified from focus group discussions

**Themes**	**Definition**	**% inter-rater agreement**
**Relative Themes**		
Genomic understanding	Relatives’ understanding of genomics	98%
Benefits/ risks of testing	Relatives’ perceived benefits and risks associated with testing	99%
Provider trust/personalized healthcare	Relatives’ trust in provider and belief provider will use testing results to guide personalized healthcare	96%
Behavioral intent to change	Relatives’ intention to change diet, exercise and screening behavior based on test results	97%
**Healthcare provider Themes**		
Genetic self-efficacy	Providers’ belief in own ability to explain genetics and test results to patients	98%
Patient wellbeing concerns	Providers’ concerns regarding patient wellbeing with testing	99%
Test validity/ clinical utility	Providers’ beliefs concerning test validity and intentions to use results to guide medical decision making for patients	98%
Belief in patient behavioral change	Providers’ belief patients will alter behavior	98%

#### ***Relative focus group discussions***

**Knowledge of genetics** Many relatives had not heard about PCa genomic testing and generally believed genetics were more important in determining PCa risk than behavior or environment. Many indicated a deterministic attitude. Relatives cited the value of PCa screening for early detection of disease but, when speaking of SNP testing, they often interchanged information obtained from screenings (i.e., detection of disease) with information obtained from genetic testing (i.e., risk of disease at a future date).

“Almost 70% of men get it anyway, and we’re double that, at least, if not triple that. So I might be negative or whatever, but I’ve come to the conclusion that I’m probably going to get it no matter what.”

“In my family it’s almost a given because there are so many people with it. It’s like, yeah, it’s part of life.”

#### Benefits and risks of testing

Relatives articulated a range of opinions regarding the benefits and risks of genomic testing. Most indicated knowing their risk would not give them additional information beyond their family history. Several indicated their belief that diet or exercise modifications would not reduce PCa risk; therefore knowing genetic susceptibility would not be useful. But other relatives noted they would be interested in knowing their own genetic risk and results might help them make more informed healthcare decisions (i.e. screenings). Relatives also expressed an interest in testing simply to satisfy their curiosity, not to necessarily use that information to make any healthcare decisions.

“It would be interesting just to know where I fell in the risk factor, having a family history of it. It would be interesting to know if I fall above, or below, or right in. It would just be interesting to know.”

Relatives also voiced concern that a lower-risk test result might give a false sense of security and lead to decreased utilization of PCa screening. Several relatives believed testing would be more beneficial to younger individuals (i.e., their sons) as younger men could then make decisions regarding earlier screening behavior. Discussions also focused on the anxiety that could be created by an increased-risk result.

“But it seems like this would be better for somebody in their twenties or thirties rather than a bunch of old guys who already know they’ve had somebody die in their family of a certain disease and can kind of look at what has happened to their ancestors.”

“If you already know that, because of your family history, you have an elevated risk of getting prostate cancer, what more is this going to tell you other than what you already know?”

“I would think this test would be more accurate because I really don’t know what my family history is. My father’s the only one that lived long enough, so I don’t know what my family history is.”

“Sometimes being ignorant and blind is better than going out there and worrying yourself to death because I have 2.5 times the risk of somebody else getting it.”

“Or if it comes back real low and gives you a false sense of security. ‘Hey, I don’t need that [to get screened]. I don’t have any problems.”

#### Provider trust and personalized healthcare

Most relatives in our study indicated they had not discussed PCa genomic testing or DTC testing with their provider. However, despite this lack of discussion, overall, relatives indicated they trusted providers to recommend genetic risk testing and to interpret the test, preferring their own provider over genetic counselors.

“Well, I’d ask him first since he’s the one I see all the time. Then if he can’t do it, he can refer me to somebody else.”

“I would [go to my internist] just … because that’s kind of where you get the information from now.”

Further, relatives indicated they expected their providers to interpret and use results to provide personalized healthcare recommendations.

“I’d want my doctor to explain it to me so I understand it fully. That would be the information I’d want.”

“And I would expect my doctor to personalize my care, directed at these higher numbers, in terms of what he’s looking for.”

“This is what it has come up with for me personally. I’d expect them to make some recommendations.”

“I would like him to give me some advice on how best I can avoid getting prostate cancer based on this.”

“This says that I was at a 35% increased risk and my lifetime risk was 22%… I would expect the doctor to be more aggressive in annual exams.”

“What my physician could do with this is, he would overlay my history and my relatives’ histories and use it as one of the factors that he would say ‘we ought to do an exam every nine months instead of every year,’ or something like that. Maybe he would increase or decrease the length between exams.”

Most relatives indicated they preferred to order the test through their provider, rather than DTC. Some were unsure whether they would go to their regular provider or to a specialist (i.e., genetic counselor) but most agreed they would consult their own provider first and follow his/her recommendations.

“I’d rather have my doctor order it because he knows exactly what to order. I wouldn’t know all the ins and outs of it.”

#### Behavioral intentions

Discussion about behavior modification following test results indicated relatives would use test results to change behaviors such as diet and exercise and would be more diligent in screening practices.

“I think it would motivate me to get tested more often, because I know I have a family history now and I don’t [get screening tests]. I get tested once every year or every two years. I would probably request to be tested more often to try to catch it earlier.”

“Yeah, maybe I’d try to eat a little better or something like that as well, but as far as the screening process goes, I’d do everything possible there is.”

As the discussion progressed, however conversations centered on behavior recommendations made in the past by providers because of other co-morbidities such as diabetes and cardiovascular disease. Relatives acknowledged they had not followed their provider’s lifestyle recommendations (i.e. lose weight, exercise more, eat a diet lower in saturated fats) and would probably not follow lifestyle behavior modification recommendations made by their provider in relation to PCa susceptibility testing.

“If it was higher, I’d go for testing, but I probably wouldn’t change how I eat because I have other reasons I should change how I eat and I didn’t. I’m miserable being fat, but it’s like a dog lying on the porch on the nail. It must not hurt it enough.”

“So have either one of us actually followed that advice? I know I haven’t.”

“I wish it were true that when I received information that I would act on it in the way I ought to, but I don’t always. I mean, you get back your cholesterol results or whatever and they aren’t in nearly such a wonderful graphical form, but you still know, ‘Oh, I need to cut down on steaks,’ or whatever. But have I? I have a father who’s died of prostate cancer, but I’m still 30 to 40 pounds overweight. So is this going to change my behavior? I wish I could say it would, but I don’t know that it’s going to.”

In addition, relatives expressed uncertainty regarding whether diet or exercise would reduce their PCa risk:

“There isn’t anything dietary you can do to prevent prostate cancer. I’ve heard that a diet high in tomatoes might help. I don’t know where I read that, but I did read it.”

“Fat men are no more likely to die of prostate cancer than skinny men or tall men or short men or what? You don’t know”.

“I didn’t think behavioral or environmental really had much of an effect on prostate cancer.”

Relatives indicated, however, that they would likely follow physician recommendations regarding PCa cancer screenings, increasing frequency or screening earlier than would otherwise be indicated by age or family history. Most relatives had favorable attitudes toward PCa testing and indicated interest in being tested.

### Providers

#### Self-efficacy in cancer genetics

 Consistent with our survey findings, focus group findings indicated providers were not confidant interpreting genetic results or explaining results to patients.

“And sometimes I have to sit and think about ..[the results] because like you said, just in that little discussion we had, we were all looking at it [statistical computation of risk]and going, now wait a minute, what is this and what is this?”

“…I hated statistics, now you are asking me to give relative risk versus lifetime risk.”

“Yeah, except when you try to explain that [the results], just think of the hard time we had just trying to get it square in our minds what all these SNPs were. Try explaining that to a patient.”

Providers also indicated they would prefer to refer patients to genetic counselors for genetic testing.

“I think I would prefer to order it though a genetic person that could help interpret, because again, then you get back to the testing, and what do you do with it?”

### Concerns regarding patient wellbeing

Similar to attitudes expressed by relatives, providers also expressed concerns that decreased-risk results would create a false sense of security and thus patients may not adhere to screening recommendations.

“What worries me is a patient that would have that kind of a family history and go pay for a genetics profile that says that they’re safe, so they decide not to go see their physician and not talk to somebody about it and put their head in the sand. That’s the patient I worry about more than somebody who comes in to talk to me.”

Most providers expressed concern that increased-risk results could increase patient anxiety and would lead to unnecessary testing and unnecessary costs to both patient and the healthcare system.

“I would wonder looking at that whole thing how many blood tests, ultrasounds, and things I’d end up doing and in this day and age of cost containment, wow. Because again, all you’re looking at are percentages. It’s not diagnosing anything. Now I have to order all those tests to rule it out.”

“…it’s like why are we doing a test that raises anxiety and increases the cost of care?”

Providers also expressed concern that test results could put patients at risk for discrimination by insurance companies and employers.

### ***Test validity/clinical utility***

Providers expressed concern regarding the validity of the PCa genomic test and overall clinical utility. Most providers would not recommend genomic PCa susceptibility testing for their patients, and did not think it would be useful in the management of patients. In general, providers strongly believed that family history was a better indicator of possible future disease.

“The problem with this is … how does that change what we already know from years and years of PSA, age, family history, all the normal clinical things…?”

Providers also indicated that they did not have sufficient time to counsel patients regarding the benefits and risks of genomic testing or interpret test results.

“To take the time to understand this report, which is not something that I’m going to have seen on a regular basis, is going to take more time than I have and I would be quite annoyed if a patient brought this in.”

“It’s a great segue into a big discussion about prostate cancer, but that’s a discussion that’s not a 10-minute discussion; it’s an hour discussion. … in real practice that’s not an easy thing to do.”

However, although providers indicated that they likely would not order the test for their patients, if a patient brought the test results to them for interpretation they would likely recommend enhanced screening practices (i.e. earlier age and/or more often than guidelines recommend) regardless of whether test results showed increased or decreased risk. Discussions focused on concerns about patient anxiety and several providers indicated that if a patient were sufficiently motivated to order the test DTC, the provider would be medically and legally obligated to recommend screening earlier or more frequently than guidelines recommend.

“Then we would probably order it [more tests] because medical/legally, if they asked for it and we didn’t order it and they happened to be the odd one that would have picked something up, I’m cooked.”

“But if they brought their test to me and they had done it because they were worried; I couldn’t just discount it. I couldn’t.”

“…. I would feel compelled to do something extra for the patient, probably screen more frequently.”

“I wouldn’t counsel somebody based on the results …. [but] I would be more vigilant about screening them if they were at higher risk.”

“…. and the only thing I would say is for the person who wants this test, I would start screening them earlier.”

### ***Belief in patient behavioral change***

Providers indicated their belief that increased-risk test results might motivate patients to adhere to screening recommendations. They also suggested that patients who are sufficiently motivated to obtain testing would also likely be highly motivated to adhere to screening recommendations.

“If you could have this test that shows a significantly higher risk of developing prostate cancer, it could be the impetus to get proper screening. If you have the reluctant patient, perhaps this test would be helpful.”

“It might actually, if a patient sees the increased risk, increase their compliance as far as coming in for regular exams or at least being willing to get the digital rectal exam.”

“I think for people who are doing it now, because they’re willing to fork over [the money] to get some information, those are the type of people I think are motivated to do something about it.”

However, providers indicated they did not believe patients would alter lifestyle behaviors (e.g., diet and exercise) based on genomic results.

“You can’t get people to do some of these things [adhere to diet and exercise recommendations] that we have known for years and have good statistical information. We can prove it over and over again how much more beneficial it would be for them and we can’t get them to do it. You get a test like this, and I don’t know if it would help or not.”

“You talk to your patient about ‘if you would lose weight your incontinence would be better, your knee arthritis would be better’; this would be better, that would be better. You wouldn’t have diabetes anymore. You wouldn’t have a risk of stroke and heart attack. You wouldn’t have to wear a CPAP. You do all those things and people still stay the same.”

## Discussion

The translation of genomic discoveries into public health practice has been slow [29-32]. At a time when the benefits of PCa screening are uncertain it is essential to understand the value of genomic information in motivating healthy behaviors and medical decision-making in persons at increased risk of the disease and providers. Since the collection of our data the U.S. Preventive Services Task Force (USPSTF) released their recommendation against PSA-based screening for PCa [6]. The American Cancer Society [5] continues to recommend men make an informed decision with their provider about whether to be screened for PCa [5]. The inconsistency regarding PCa screening makes medical decision-making in this context even more challenging for both consumers and providers. Understanding how both consumers and providers regard PCa susceptibility testing can help determine how to best formulate decision support tools and interventions to guide appropriate healthcare decisions. Yet, while prior studies have examined consumers’ [33-35] and providers’ [16,18,36] knowledge and attitudes regarding general DTC genetic testing, our study is among the first to specifically examine both at-risk men’s and providers’ attitudes and knowledge regarding PCa susceptibility testing. Thus, our study’s findings provide a unique contribution to the literature regarding the knowledge, attitudes and behavioral intentions in both men at familial risk of PCa and in providers most likely to order or interpret genetic testing for PCa susceptibility.

Prior studies have reported low levels of awareness of DTC testing [37-39]. Our findings support these studies, documenting low levels of awareness about genetic testing in general and PCa susceptibility testing in particular. Similar to relatives, and also supporting prior research [16], primary care providers in our study had low levels of awareness of DTC genomic testing. Moreover, urologists in our study exhibited low levels of awareness with only half indicating they had heard or read about DTC genomic tests. It is noteworthy that relatives in our study and participants in prior studies [22] have indicated that they prefer to obtain and discuss genetic testing through their own provider, yet both primary care physicians and urologists, the two providers most likely to provide medical advice to men at increased PCa familial risk exhibit low levels of awareness about this type of genomic testing. This limited awareness as well as low levels of genomic efficacy could impact patient-provider communication about PCa susceptibility testing.

We observed appreciable differences in relatives’ and providers’ attitudes toward PCa genomic testing. Providers identified limitations of the genomic risk panel and expressed concerns about the test’s clinical validity and utility. The majority indicated they would not order the test for their patients and would not use PCa genomic testing to guide healthcare decisions, preferring to use family history when making screening recommendations for their patients. However, while relatives may not have found testing to be helpful in making lifestyle choices (i.e., diet, exercise), most relatives in our study indicated interest in testing, both to understand genomic factors that may help with screening decision-making and also simply out of curiosity. In fact, many relatives in our study indicated they would get the test simply because it would be interesting. These findings also support prior research on public interest in genomic testing interest [35]. Despite citing curiosity as a reason for testing, relatives expected their provider to interpret the results and make healthcare recommendations based on their test results. Relatives in our study also indicated that they trusted their providers could and would accurately interpret the test results, and most would seek out their own provider for information to help them make an informed decision about testing, rather than seeing a genetic specialist. However, primary care physicians and general practitioners have consistently reported low genomic self-efficacy [16,18]. Providers in our study similarly indicated low confidence in discussing benefits and risks of genetic risk testing with patients. Furthermore, many providers in our study indicated they did not have time to counsel patients about genetic issues, and would prefer to send patients to a genetic counselor for testing and have them interpret the test results. Providers were concerned that decreased-risk results would lead to less compliance with screening recommendations. They also believed that increased-risk results would lead to greater anxiety and unnecessary procedures which in turn could lead to unnecessary costs to the patient and to the healthcare system. Additionally, most providers in our study had not encountered patients’ questions regarding DTC testing. Similar findings are reported in the literature, [16] suggesting that providers may lack confidence because they lack experience. This lack of experience and self-efficacy could influence providers’ ability to help patients make informed decisions about testing and how to cope with the test results.

While relatives’ expectations of providers in our study are consistent with patient expectations in prior research [35], many relatives also indicated that although they would consider enhanced screening, they did not think genomic information added value to family history information. This contradicts other studies examining public attitudes toward genomic testing [39]. One possible explanation for this attitude may be the relatives’ belief that neither diet nor exercise could reduce their PCa risk despite scientific evidence suggesting some associations between diet, exercise and risk of PC development [40-44]. If individuals believe there are steps they can take to eliminate or reduce a risk, then information about that risk can be advantageous. For instance, if individuals receive genetic cancer risk information related to increased risk for skin melanoma and they believe they can take steps to reduce the risk through behavior change (i.e. limit sun exposure; wear sunscreen), then genetic risk information may demonstrate clinical utility [45]. However, while relatives indicated their belief that healthy behaviors could reduce the risk of disease for people who have a gene for a particular disease they also indicated they did not believe lifestyle changes would reduce their own PCA risk. Thus they may have felt that genetic information would not help them make lifestyle behavior changes that would be beneficial.

We observed similarities in providers and relatives attitudes toward lifestyle behavior change. Relatives indicated they would follow enhanced screening recommendations based on risk results but admitted they might not follow through if their provider made lifestyle recommendations for diet and exercise behavior changes. Providers indicated they would recommend enhanced screening for patients who had received genetic risk testing and also believed relatives would likely not make diet and exercise changes.

Noteworthy is the finding that while providers believed family history was sufficient for making screening recommendations, most relatives had not had a conversation with their own provider about their PCa family history. Further, nearly one-third of relatives indicated they had initiated conversations with providers regarding PCa screening because the provider had not initiated the conversation. This is informative, as the new USPSTF guidelines recommend against PSA testing but recognize the common use of PSA screening in practice today. The guidelines suggest that patients requesting PSA screening be provided with opportunities to make informed choices; thus providers should be prepared to discuss screening benefits and risks. Our study appears to support the notion that while providers may not recommend PSA testing they may be asked to provide screening tests as most relatives in our study had their PSA levels tested within the prior year despite the lack of patient/provider communication about this issue.

Another important finding in our study concerns overutilization of health services. As discussed, providers have low levels of confidence in providing genetic services and indicated they would not order testing for their patients. Yet if patients presented them with their test results, providers indicated they would recommend enhanced PCa screening *regardless of test result outcomes*. Providers stated that if a patient was sufficiently concerned to order a test, then the physician would increase screening recommendations. There is considerable controversy surrounding PCa screening including over-diagnosis and over-treatment of clinically indolent cancers that can result in treatment-related side effects and incur unnecessary healthcare costs and burdens on the healthcare system [46]. Concerns have been raised that consumer use of genomic testing may lead to over utilization of health care systems. Our results indicate this may occur not because physicians believe the test results indicate the need for more diligence in patient surveillance, but because physicians worry about medical and legal responsibility if they do not adequately address test results with increased screening. Our findings underscore the need for educating both consumers and providers regarding genetic testing based on an understanding of both perspectives. Decision-support tools are vital to both patients and providers to ensure that patients are made aware of the risks and limitations of genetic testing and providers make healthcare decisions for their patients based on their patients’ clinical characteristics.

It should be noted that this was a pilot study and as such our sample was fairly small and homogenous: relatives were primarily non-Latino white and well educated with higher incomes. In addition, we drew our sample from one geographical area and relatives’ attitudes and knowledge might not be generalizable to other geographical areas, or to lower education or income levels. Provider’s genetic awareness and knowledge might also differ by geographical location. It is also worth noting that the relatives in our study consisted of men who were aware of their increased risk for PCa via their own family history. It could be that their awareness of this risk influenced their interest in testing. Future research should concurrently examine testing interest in men with and without family histories of PCa.

Additionally, the education session was created to provide a minimal level of information and was presented without bias toward or against SNP testing. We did not measure genomic knowledge levels in the post-focus group surveys, thus we could not determine whether the education session had changed genetic understanding and thus testing intentions. It would be of value for future research to include measures that assess changes in genetic understanding following focus group discussions.

## Conclusion

Our study provides novel information about the attitudes and intentions of individuals at high-risk for PCa, as well as providers who may order, interpret or use results from PCa susceptibility tests in clinical decision making for these particular individuals. Our findings also demonstrate the value of examining both patient and provider attitudes, knowledge and behavioral intentions. An understanding of both patient and provider similarities and differences may influence the effective translation of novel genomic tests into clinical practice. Our results are consistent with previous studies about DTC testing in the general public, and further serve to document the need for decision-support tools for both consumers of genomic testing and health care providers.

## Endnotes

^a^Participants were not offered DTC testing as part of this study.

^b^deCODE Genetics is no longer offering DTC genetic testing.

## Competing interests

The author declare that they have no competing interests.

## Authors’ contributions

NA, WK, LA, JB, CD and AK were involved in conceiving the study. AK, WCB, WK and LGA were involved in designing the surveys, the focus group presentation and moderator guide. NA WCB, JB and MW were responsible for participant recruiting. WCB, WK and MW facilitated the focus groups and semi-structured interviews. WCB and MW coded the focus groups and all authors read the manuscripts and contributed to data analysis. WCB led the writing process and all authors commented on sequential drafts and approved the final version of the manuscript.

## Pre-publication history

The pre-publication history for this paper can be accessed here:

http://www.biomedcentral.com/1472-6963/13/279/prepub

## Supplementary Material

Additional file 1 Survey measures. Click here for file

Additional file 2 Education Sessions. Click here for file
