# Supplementary material for: Patient and provider attitudes toward genomic testing for prostate cancer susceptibility: a mixed method study
Source: BMC Health Serv Res. 2013 Jul 20;13:279. doi: 10.1186/1472-6963-13-279 (PMC3750463; doi:10.1186/1472-6963-13-279)
Supplement: Additional file 1 — Survey measures. [file 1472-6963-13-279-S1.doc]

**Appendix A.** Survey measures

| **Survey items** | **Time administered** | **Style** | **Description** |
| --- | --- | --- | --- |
| **Relatives** |  |  |  |
| Demographics | Baseline | Check box | We collected information on race, marital status, age, education, employment status and household income. |
| Awareness | Baseline | Yes/no | Awareness of DTC genetic testing was measured with the single (yes/no) item from the State Behavioral Risk Factor Surveillance System and National Health Styles Surveys on Awareness and Use of Direct-to-Consumer Nutrigenomic Tests . |
| Prostate cancer screening intentions  and behavior | Baseline | Self report | Items were adapted from measures developed for the National Cancer Institute assessed screening history, screening adherence, and plans for future PCa screening. |
| Perceived risk | Baseline | 5-point Likert scale; very small to very great. | A four-item measure asked relatives to rate their perceived susceptibility to prostate cancer. This previously validated scale has been found to have good internal reliability (Cronbach’sα=.73) . |
| Perceived worry | Baseline | 5-point Likert scale; never or not at all to all the time or extremely | This 3-item measure assessed intensity and frequency of PCa worry. Participants were asked how often they worry about developing PCa in their lifetime and how bothered or how worried they are about developing the disease [4]. |
| Genetic opinions, attitudes & knowledge | Baseline | 5-point Likert scale; strongly disagree to strongly agree | Two measures assessed genetic understanding, opinions and attitudes. The Gene-Behavior Interaction Beliefs Scale consists of 8 items and has demonstrated high reliability (α=.8). A second measure consisted of nine items which assessed beliefs about genetic causation and its modifiability through healthy or unhealthy behaviors for disease in general. Items for this measure were adapted from Lynch et al., 2008 , Parrott et al., 2004 and from personal communications between one of the study authors (LGA) and CM Condit. New items created for the present study assess offsetting effects of healthy behavior. |
| Testing intentions | Post | Yes/no/unsure | Intention to obtain PCa SNP testing was assessed with a single item, “Do you think you would be likely to get a genetic assessment test for prostate cancer risk?” Relatives were also asked whether they would prefer to order the test on their own or through their provider. |
| Presentation opinions | Post | Free text | Relatives were asked to provide free text responses to items assessing what they liked and didn’t like about the educational presentation, what was easy to understand, what was hard to understand, as well their preferences for obtaining genetic information (i.e., web pages, interactive CD, one-on-one sessions). Participants also listed additional information they would want presented. |
| **Healthcare provider** |  |  |  |
| Demographics | Baseline | Check box | Baseline survey items assessed providers’ age, years in practice, specialty and practice setting (i.e., primary care, community urology, academic urology). |
| Attitudes | Baseline | 5-point Likert scale; strongly disagree to strongly agree. | An adapted 7-item measure was used to assess providers’ attitudes and opinions regarding the clinical usefulness of genetic testing as well as perceived benefits and perceived concerns of genetic testing . A six-item adapted measure evaluated attitudes and opinions with items such as “It is important for me to learn about new advances in genetics” and “I have sufficient time in my practice to counsel patients about genetic risk.” |
| Awareness (yes/no) | Baseline | Yes/no | Providers’ awareness of genetic DTC testing was assessed with a single item (yes/no) asking if they had ever hear or read about the genetic tests. |
| Experience with patients | Baseline | Yes/no and % of patients | Eight adapted questions assessed whether providers who were aware of DTC testing had ever discussed DTC tests with a patient or had a patient bring in results of DTC genetic tests. |
| Genomic self-efficacy | Baseline | 5-point Likert scale; low to high confidence | We assessed providers’ confidence in their ability to carry out core clinical genetic competencies in the delivery of genetic services across several domains: eliciting genetic information, assessing risk of hereditary disorders, deciding who should be offered referral for genetic counseling or testing, ordering genetic testing, evaluating and discussing clinical usefulness of a genetic test, providing counseling on genetic decision-making, providing psychosocial support following test results and overall confidence in cancer genetics **.** |
| Testing intentions | Post | Free text | Assessment of providers’ intentions regarding PCa SNP testing for their patients was measured with a single item (yes/no) item, “After viewing the presentation today, do you think you would be likely to recommend genetic risk assessment tests for your patients?” Providers could provide free text responses regarding why or why they would not recommend testing. |
| Clinical usefulness | Post | 4-point Likert scale; very useful to not at all useful | Attitudes toward clinical usefulness were assessed with a single-item, “How useful would single nucleotide polymorphism (SNP)-based assessment be in the management of patients?” |
| Presentation opinions | Post | Free text | Providers were asked to provide free text responses to items assessing what they liked and didn’t like about the educational presentation as well their preferences for obtaining genetic information (i.e., web pages, interactive CD, one-on-one sessions) and to specify what additional information they would want presented. |

References

1. Goddard KA, Duquette D, Zlot A, Johnson J, Annis-Emeott A, Lee PW, Bland MP, Edwards KL, Oehlke K, Giles RT, *et al*: **Public awareness and use of direct-to-consumer genetic tests: results from 3 state population-based surveys, 2006.** *Am J Public Health* 2009, **99**(3)**:**442–445.

2. Vernon SW, Meissner H, Klabunde C, Rimer BK, Ahnen DJ, Bastani R, Mandelson MT, Nadel MR, Sheinfeld-Gorin S, Zapka J: **Measures for ascertaining use of colorectal cancer screening in behavioral, health services, and epidemiologic research.** *Cancer Epidemiol Biomarkers Prev* 2004, **13**(6)**:**898–905.

3. Kremers SP, Mesters I, Pladdet IE, Van Den BB, Stockbrugger RW: **Participation in a sigmoidoscopic colorectal cancer screening program: a pilot study.** *Cancer EpidemiolBiomarkers Prev* 2000, **9**(10)**:**1127–1130.

4. *Worry.* http://dccps.cancer.gov/brp/constructs/worry/w6.html.

5. Condit CM, Shen L: **Public understanding of risks from gene-environment interaction in common diseases: implications for public communications.** *Public Health Genomics* 2011, **14**(2)**:**115–124.

6. Lynch J, Bevan JL, Jennifer L, Achter P, Harris T, Condit CM: **A preliminary study of how multiple exposures to messages about genetics impact on lay attitudes towards racial and genetic discrimination.** *New Genetics and Society* 2008, **27**(1)**:**43–56.

7. Parrott R, Silk K, Raup Krieger J, Harris T, Condit C: **Behavioral health outcomes associated with religious faith and media exposure about human genetics.** *Health Commun* 2004, **16**(1)**:**29–45.

8. Carroll JC, Rideout AL, Wilson BJ, Allanson JM, Blaine SM, Esplen MJ, Farrell SA, Graham GE, MacKenzie J, Meschino W, *et al*: **Genetic education for primary care providers: improving attitudes, knowledge, and confidence.** *Can Fam Physician* 2009, **55**(12)**:**e92–99.

9. Powell KP, Cogswell WA, Christianson CA, Dave G, Verma A, Eubanks S, Henrich VC: **Primary care physicians' awareness, experience and opinions of direct-to-consumer genetic testing.** *J Genet Couns* 2012, **21**(1)**:**113–126.
