# Supplementary material for: Patient and provider attitudes toward genomic testing for prostate cancer susceptibility: a mixed method study
Source: BMC Health Serv Res. 2013 Jul 20;13:279. doi: 10.1186/1472-6963-13-279 (PMC3750463; doi:10.1186/1472-6963-13-279)
Supplement: Additional file 2 — Education Sessions. [file 1472-6963-13-279-S2.doc]

**Appendix B**

**Table 1**. Information presented to relatives in focus group presentation

| **Prostate Cancer** | **Screening** | **SNP testing** |
| --- | --- | --- |
| Occurrence rates | Recommended approaches: prostate specific antigen (PSA); digital rectal exam (DRE) | Genes as instructions; genetic code (A, T, C, G) |
| Diagnosis rates | Recommendations to begin screening based on age and family history | Variation in genetic code may lead to differences in physical features; some may have no effect; some cause a gene not to function and cause certain diseases. These are called mutations |
| Mortality rates | Benefits of prostate cancer (PCa) screening | Mutations may cause very high risk for certain cancers |
| Risk factors | Limitations of PCa screening | No high-risk PCa genes have currently been identified |
|  |  | SNPs are changes that affect one letter of the genetic code; when evaluated together, they may indicate increased or decreased disease risk |
|  |  | New test available direct to consumer or physician ordered; may provide personalized PCa risk |
|  |  | Sample deCODE test result shown |
|  |  | SNPs can also evaluate risk for hundred of diseases and physical traits |
|  |  | Benefits and limitations of SNP testing for PCa susceptibility; options to collect sample at home |

**Table 2**. Information presented to providers in focus group presentation

| **Prostate Cancer** | **Screening** | **SNP testing** |
| --- | --- | --- |
| Age and race as risk factors | Recommended approaches (PSA; digital rectal exam) | Genes associated with cancer predisposition: BRCA1/BRCA2; MLH1/MSH2/MSH6/PMS2; CDKN2A |
| Family history as risk factor | Recommended ages to begin screening based on age and family history | No PCa susceptibility gene has been identified |
|  |  | Single-nucleotide polymorphisms: single base change in DNA sequence; common in genome; |
|  |  | Case-control studies identified several SNPs found more commonly in men who develop PCa compared to men who do not |
|  |  | Table of relative risks associated with individual SNPs; relative risks are combined for an overall relative risk (RR) |
|  |  | Limitations of SNP testing; cost of SNP testing |
|  |  | Sample deCODE test result shown |
|  |  | Benefits/limitations of SNP testing for PCa susceptibility |
